# Fuzheng Huayu recipe prevents nutritional fibrosing steatohepatitis in mice

**DOI:** 10.1186/1476-511X-11-45

**Published:** 2012-03-28

**Authors:** Yan-Hong Jia, Rong-Qi Wang, Hong-Mei Mi, Ling-Bo Kong, Wei-Guang Ren, Wen-Cong Li, Su-Xian Zhao, Yu-Guo Zhang, Wen-Juan Wu, Yue-Min Nan, Jun Yu

**Affiliations:** 1 Department of Traditional and Western Medical Hepatology, Third Hospital of Hebei Medical University, Shijiazhuang, China; 2 Institute of Digestive Disease and Department of Medicine and Therapeutics, Li KaShing Institute of Health Sciences, The Chinese University of Hong Kong, Hong Kong, China

**Keywords:** FuzhengHuayu, Chinese herb, Non-alcoholic steatohepatitis, Hepatic fibrosis, Oxidative stress

## Abstract

**Background:**

Fuzheng Huayu recipe (FZHY), a compound of Chinese herbal medicine, was reported to improve liver function and fibrosis in patients with hepatitis B virus infection. However, its effect on nutritional fibrosing steatohepatitis is unclear. We aimed to elucidate the role and molecular mechanism of FZHY on this disorder in mice.

**Methods:**

C57BL/6 J mice were fed with methionine-choline deficient (MCD) diet for 8 weeks to induce fibrosing steatohepatitis. FZHY and/or heme oxygenase-1 (HO-1) chemical inducer (hemin) were administered to mice, respectively. The effect of FZHY was assessed by comparing the severity of hepatic injury, levels of hepatic lipid peroxides, activation of hepatic stellate cells (HSCs) and the expression of oxidative stress, inflammatory and fibrogenic related genes.

**Results:**

Mice fed with MCD diet for 8 weeks showed severe hepatic injury including hepatic steatosis, necro-inflammation and fibrosis. Administration of FZHY or hemin significantly lowered serum levels of alanine aminotransferase, aspartate aminotransferase, reduced hepatic oxidative stress and ameliorated hepatic inflammation and fibrosis. An additive effect was observed in mice fed MCD supplemented with FZHY or/and hemin. These effects were associated with down-regulation of pro-oxidative stress gene cytochrome P450 2E1, up-regulation of anti-oxidative gene HO-1; suppression of pro-inflammation genes tumor necrosis factor alpha and interleukin-6; and inhibition of pro-fibrotic genes including α-smooth muscle actin, transforming growth factor beta 1, collagen type I (Col-1) and Col-3.

**Conclusions:**

Our study demonstrated the protective role of FZHY in ameliorating nutritional fibrosing steatohepatitis. The effect was mediated through regulating key genes related to oxidative stress, inflammation and fibrogenesis.

## Background

Non-alcoholic steatohepatitis (NASH) represents the most common chronic liver disease associated to environment and lifestyle conditions in a context of genetic predisposition. It has become one of the leading causes of severe hepatic dysfunction in the modern world [1]. Liver fibrosis, through which NASH develops into cirrhosis, is a healing response to chronic injuries. Fibrogenesis involves multifactorial oxidative stress, cytokine imbalance, and hepatic stellate cells (HSCs) activation. Unfortunately, today there is no specific and effective antifibrotic therapy available, therefore it is rather important to recognize liver fibrosis in its early stages and search for new treatment method to prevent further progression.

Fuzheng Huayu recipe (FZHY), a compound of Chinese herbal medicine, consists of six Chinese medicinal herbs, namely Semen Persicae, Radix Salvia Miltiorrhizae, Gynostemma Pentaphyllammak, Cordyceps, Pollen Pini, Fructus Schisandrae Chinensis [2]. The previous clinical trials showed that FZHY could significantly improve clinical symptoms, liver function, reverse hepatic fibrosis and decrease portal pressure in patients with chronic hepatitis B and liver cirrhosis [3,4]. However, the effect of FZHY on fibrosing steatohepatitis remains unknown. In this study, we investigated the role and molecular basis of FZHY in the evolution of nutritional fibrosing steatohepatitis in mice.

## Material and methods

### Animals and treatments

Eight-week-old male C57BL/6 J mice with body weight between 20-25 g were obtained from the Experimental Animal Center of Chinese Academy of Medical Sciences, and were bred in a temperature-controlled animal facility with a 12-h light-dark cycle. They had free access to water and were allowed to adapt to their food and environment for 1 week before the start of the experiment. The C57BL/6 J mice were randomly divided into 5 groups (6 mice per group): 1) MCD group, mice fed methionine-choline deficient diet (ICN, Aurora, Ohio); 2) control group, mice fed MCD diet supplemented with choline bitartate (2 g/kg) and DL-methionine (3 g/kg) (ICN, Aurora); 3) MCD + hemin group, mice fed MCD diet administered with heme oxygenase-1 (HO-1) chemical inducer hemin (30 μmol/kg) by intraperitoneal (i.p.) injections three times per week; 4) MCD + FZHY group, mice fed MCD diet supplemented with FZHY (15 g/kg^.^d, Huanghai pharmaceutical company limited, Shanghai, China); 5) MCD + FZHY + hemin group, mice fed MCD diet administered with FZHY and hemin. The duration of the experiment is up to 8 weeks. During the experiments, their body-weight and rate of diet consumption were recorded. All of the animals were sacrificed after overnight fasting at the end of experiments. Blood samples were collected from femoral artery for biochemical analysis. Livers were weighed and fixed in 10% formalin for histological analysis or snap-frozen in lipid nitrogen followed by storage at -80°C freezer until required. All the protocols and procedures were performed following the guidelines of the Hebei Committee for Care and Use of Laboratory Animals and were approved by the Animal Experimentation Ethics Committee of the Hebei Medical University.

### Biochemical analysis

Serum alanine aminotransferase (ALT) and aspartate aminotransferase (AST) levels were measured by the enzymatic kinetic method using an automatic biochemical analyzer (Olympus UA2700, Japan) according to the manufacturer's instructions. The extent of lipid peroxidation in the liver homogenate was estimated by measuring the concentration of malondialdehyde (MDA) using an OxiSelect thiobarbituric acid-reactive substances (TBARS) Assay Kit according to the manufacturer's instructions (Cell Biolabs, San Diego, CA).

### Histological analysis

Hematoxylin and eosin stained and Masson trichromatism stained paraffin-embedded liver sections (5 μm thick) were scored for hepatic steatosis, inflammation and fibrosis as described previously [5,6] in accordance with the Brunt's criteria [7] and the histological scoring system for NAFLD issued by the Pathology Committee of the Nonalcoholic Steatohepatitis Clinical Research Network [8].

### Quantitative real-time reverse transcription polymerase chain reaction (RT-PCR) analysis of hepatic messenger RNA expression

Total RNA was isolated from frozen liver tissues using Trizol Reagent (Invitrogen, Carlsbad, CA) according to the manufacturer's instructions. The hepatic messenger RNA (mRNA) levels of cytochrome P450 2E1 (CYP2E1), HO-1, tumor necrosis factor alpha (TNF-α), interleukin-6 (IL-6), α-smooth muscle actin (α-SMA), transforming growth factor beta 1 (TGF-β1), collagen type I (Col-1) and Col-3 were determined by quantitative Real-Time reverse transcription polymerase chain reaction (qRT-PCR) using the ABI PRISM 7300 sequence detection system (Applied Biosystems, Foster, CA) with SYBR Green Reagent (Invitrogen, Carlsbad, CA). Expression levels of the target genes were normalized against an endogenous reference gene glyceraldehydes 3-phosphate dehydrogenase (GAPDH). The specific primers for CYP2E1, HO-1, TNF-α, IL-6, α-SMA, TGF-β1, Col-1 and Col-3 were designed using Primer Express 2.0 (Table 1). All data were obtained using Sequence Detector Software (Applied Biosystems, Foster, CA).

**Table 1:** Primers for real-time quantitative PCR analysis

Gene	Product length	Primer sequences
HO-1	427 bp	F 5'-AACAAGCAGAACCCAGTCTATG-3'

		R 5'-TGAGCAGGAAGGCGGTCTTA-3'

CYP2E1	199 bp	F 5'-AACAGAGACCACCAGCACA-3'

		R 5'-GGAAGGGACGAGGTTGATGA-3'

TNF-α	79 bp	F 5'-GAACTGGCAGAAGAGGCACT-3'

		R 5'-AGAAGAGGCTGAGACATAGGC-3'

IL-6	85 bp	F 5'-TACCACTCCCAACAGACCTG-3'

		R 5'-TCTCATTTCCACGATTTCCCAG-3'

α-SMA	162 bp	F 5'-ATGATGCTCCCAGGGCTGTT -3'

		R 5'-TGGTGATGATGCCGTGTTCT-3'

TGF-β1	85 bp	F 5'-CCGCAACAACGCCATCTATG -3'

		R 5'-TGCTTCCCGAATGTCTGACG-3'

Col-1	108 bp	F 5'-TGACTGGAAGAGCGGAGAGT-3'

		R 5'-GTAGGGAACACACAGGTCTGA-3'

Col-3	61 bp	F 5'-CTGGTGCTAAGGGTGAAGTTG-3'

		R 5'-TGTCCTGGTGAGCCATTTGAG-3'

GAPDH	120 bp	F 5'-TGAACGGGAAGCTCACTGG-3'

		R 5'-GCTTCACCACCTTCTTGATGTC-3'

Abbreviations: *HO-1 *heme oxygenase-1, *CYP2E1 *cytochrome p4502E1, *TNF-α *tumor necrosis factor-alpha, *IL-6 *interleukin-6, *α-SMA *α-smooth muscle actin, *TGF-β1 *transforming growth factor beta 1, *Col-1 *collagen type I, *Col-3 *collagen type III, *GAPDH *glyceraldehyde 3-phosphate dehydrogenase

### Western blotting analysis of hepatic protein expression

Total protein was extracted and concentration was measured by the Bradford method (DC protein assay; Bio-Rad, Hercules, CA) as previously described [9]. Equal amounts of protein (100 μg/well) were loaded onto 10% SDS-PAGE for each sample and proteins were transferred onto equilibrated polyvinylidene difluoride membranes (Amersham Biosciences, Buckinghamshire, UK) by electroblotting. The membranes were incubated with primary antibodies of CYP2E1, HO-1, α-SMA, TGF-β1, Col-1 and Col-3 (Santa Cruz Biotechnology, Santa Cruz, CA) respectively overnight at 4°C. Membranes were further incubated with secondary antibody for 1 h at room temperature. Proteins were detected by enhanced chemiluminescence (ECL; Amersham Corporation, Arlington Heights, CA) and bands were quantified via scanning densitometry using the digital Kodak Gel Logic 200 (Carestream Molecular Imaging, Newhaven, CT). Individual levels of hepatic protein were normalized with β-actin.

### Statistical analysis

All data are presented as mean ± SD (standard deviation). Statistical analysis was performed by one-way analysis of variance (ANOVA) and Student-Newman-Keuls test for evaluating differences between groups using SPSS 13.0 (v. 13.0; SPSS Inc., Chicago, IL). A *P-*value of less than 0.05 was considered statistically significance.

## Results

### FZHY lowered the serum levels of ALT and AST in mice fed with MCD diet

**Figure 1:**
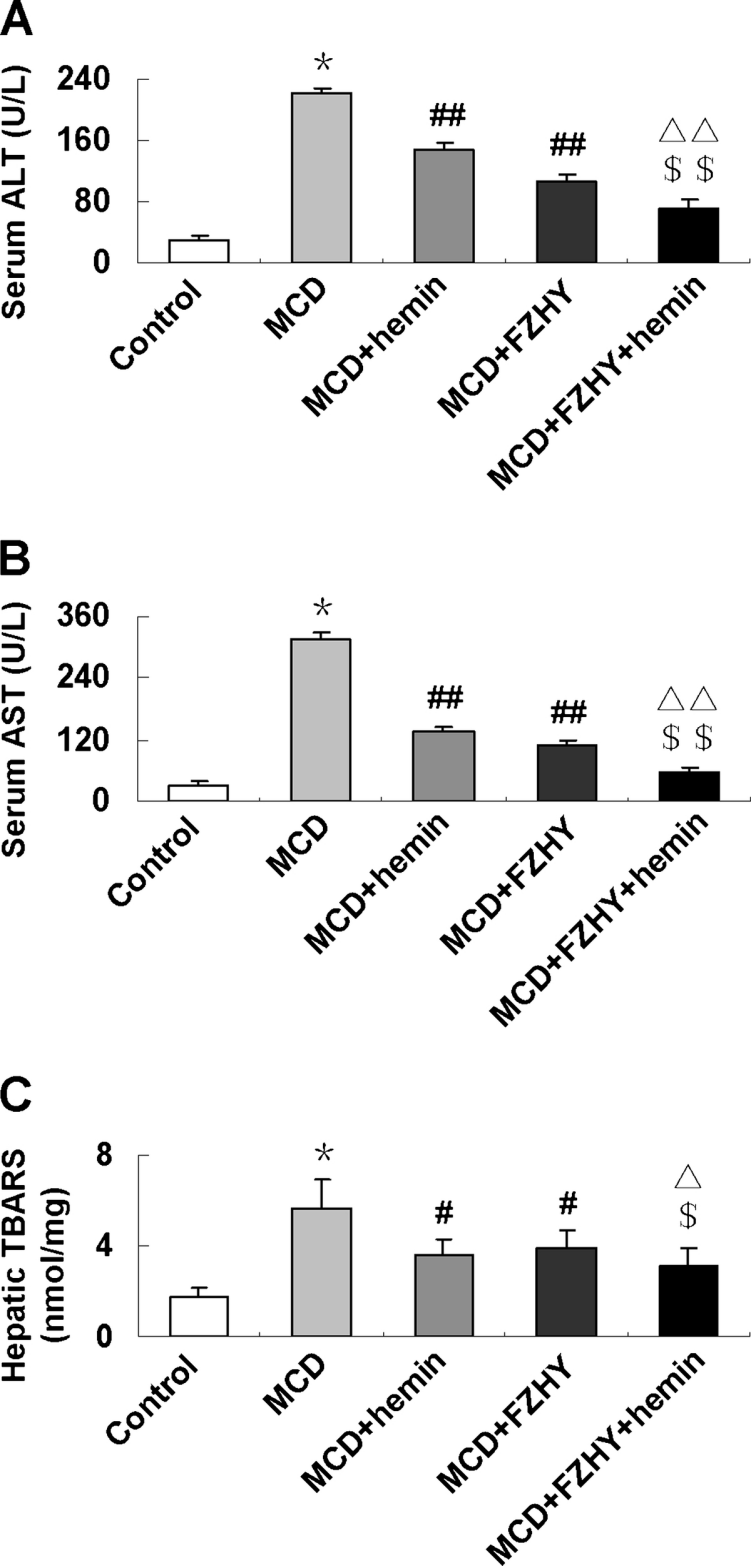
**Effect of the MCD diet and treatment with FZHY and hemin on: (A) Serum alanine aminotransferase (ALT); (B) Serum aspartate aminotransferase (AST); (C) Hepatic malondialdehyde (MDA) content**. Data are expressed as the mean ± SD (n = 6 per group). *P < 0.001, compared with control group; ^#^*P *< 0.05, ^##^*P *< 0.01, compared with MCD group; ^$^*P *< 0.01, ^$$^*P *< 0.001, compared with MCD + FZHY group; ^Δ^*P *< 0.05, ^ΔΔ^*P *< 0.01, compared with MCD + Hemin group.

As shown in Figure 1, mice fed an MCD diet showed significantly higher serum ALT and AST levels (*P *< 0.001) compared with control group, indicating hepatic injury. A significant reduction of serum ALT and AST (*P *< 0.001) were noticed after FZHY with or without hemin administration. An additive effect was observed in the mice treated with FZHY and hemin. In addition, FZHY lowered hepatic oxidative stress as demonstrated by TBARS assay, the combination of FZHY and hemin showed a better effect on suppressing MDA concentrations (Figure 1C).

### Effect of FZHY on hepatic inflammation and fibrosis in mice fed with MCD diet

**Figure 2:**
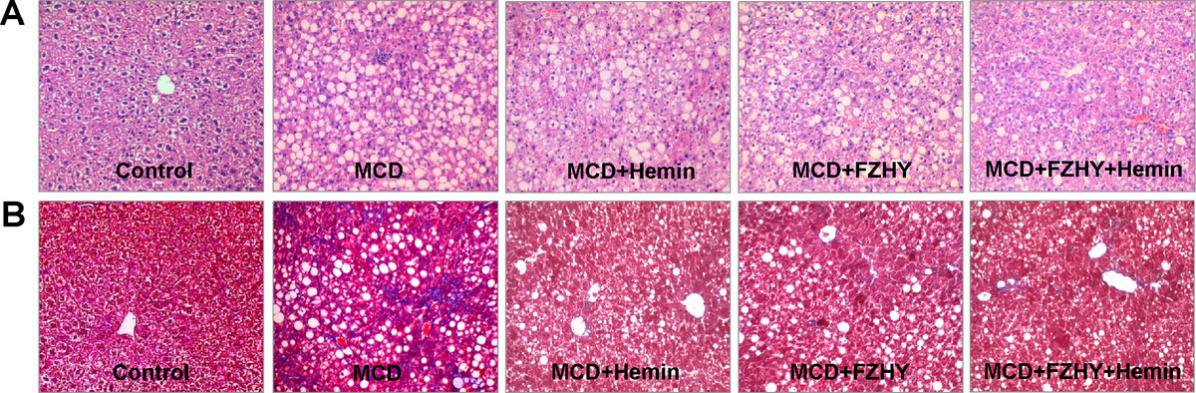
**Histopathological changes of liver sections in mice under various treatment conditions**. Hematoxylin and eosin stained (A) and Masson trichromatismstained (B) liver sections from mice liver (Original magnification, ×200)

The liver sections from mice fed an MCD diet alone exhibited disordered lobule structure, severe macrosteatosis, spot or focal hepatocyte necrosis and inflammatory infiltration (Figure 2A), portal fibrosis and fibrous septum (Figure 2B). However, mice treated with FZHY in the presence or absence of hemin could notably ameliorate hepatic steatosis, necrotic inflammation (Figure 2A) and improved liver fibrosis (Figure 2B). Co-administration of FZHY and hemin had a further improved effect on hepatic inflammation and fibrosis (Figure 2B).

### Effect of FZHY on the expression of oxidative related genes CYP2E1 and HO-1

**Figure 3:**
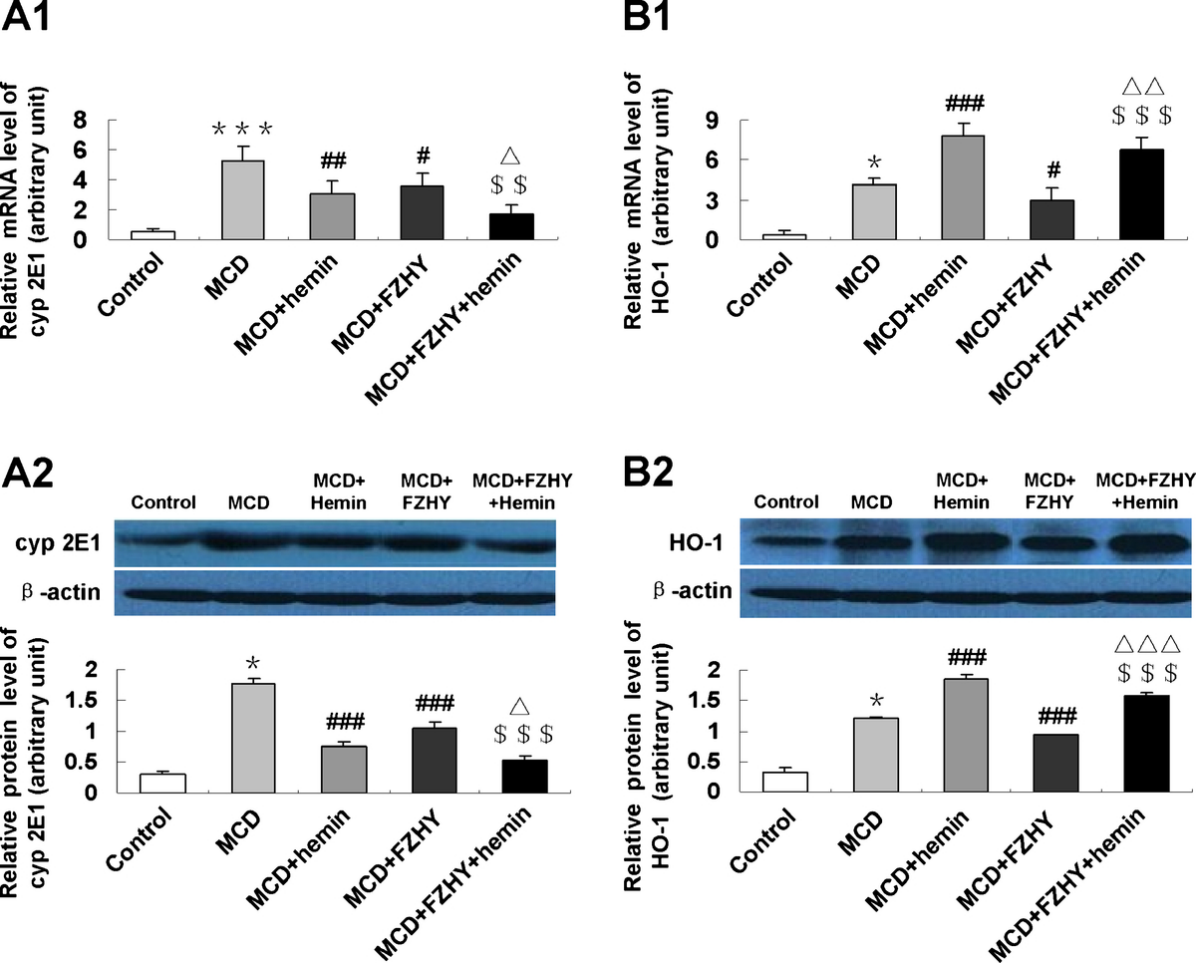
**Effect of FZHY and hemin on hepatic CYP2E1 and HO-1 expression**. mRNA expression of CYP2E1 (A1) and HO-1 (B1) were determined by quantitative real-time PCR; protein levels of CYP2E1 (A2) and HO-1 (B2) were detected by Western blot. Data are expressed as the mean ± SD (n = 6 per group). *P < 0.05, *** *P *< 0.001, compared with control group; ^#^*P *< 0.05, ^##^*P *< 0.01, ^###^*P *< 0.001, compared with MCD group; ^$$^*P *< 0.01, ^$$$^*P *< 0.001, compared with MCD + FZHY group; ^Δ^*P *< 0.05, ^ΔΔ^*P *< 0.01, ^ΔΔΔ^*P *< 0.001, compared with MCD + Hemin group.

The mRNA and protein expressions of lipid peroxidation mediator CYP2E1 and anti-oxidative stress factor HO-1 were induced by MCD diet (Figure 3A and 3B). Administration of FZHY could reduce hepatic expression of CYP2E1 and HO-1, which was concomitant with ameliorated hepatic steatosis, inflammation and fibrosis induced by MCD-treatment. A further inhibition of CYP2E1 expression was observed in FZHY plus hemin group.

### FZHY regulated expression of inflammation related genes

**Figure 4:**
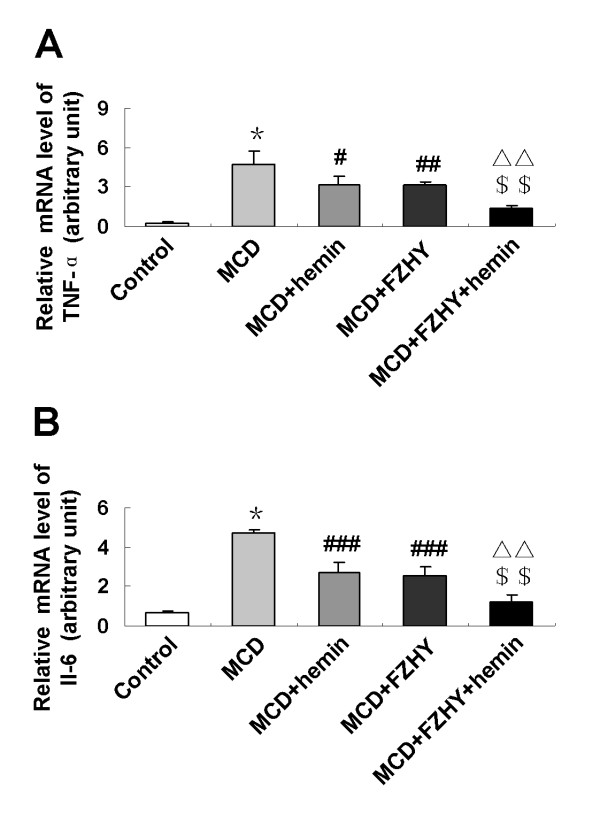
**Effect of FZHY and hemin on expression of hepatic inflammatory factors in MCD diet-induced fibrosing steatohepatitis**. mRNA expression of TNF-α (A), IL-6 (B) were determined by quantitative real-time PCR. Data are expressed as the mean ± SD (n = 6 per group). *P < 0.001, compared with control group; ^#^*P *< 0.05, ^##^*P *< 0.01, ^###^*P *< 0.001, compared with MCD group; ^$$^*P *< 0.01, ^$$$^*P *< 0.001, compared with MCD + FZHY group; ^ΔΔ^*P *< 0.01, compared with MCD + Hemin group.

To seek an explanation for the decreased serum transaminases and ameliorated liver histology by FZHY, we investigated hepatic mRNA expression levels of inflammatory genes TNF-α and IL-6. Relative to control mice, hepatic TNF-α and IL-6 were up-regulated in MCD diet-fed mice (*P *< 0.001) (Figure 4), which was significantly blunted by treatment with FZHY or hemin. The combination of FZHY and hemin led to a further effect on preventing the TNF-α (*P *< 0.01) and IL-6 (*P *< 0.01) mRNA expression compared with MCD + hemin group.

### FZHY suppressed hepatic expression of pro-fibrosis genes in MCD diet-induced fibrosing steatohepatitis

**Figure 5:**
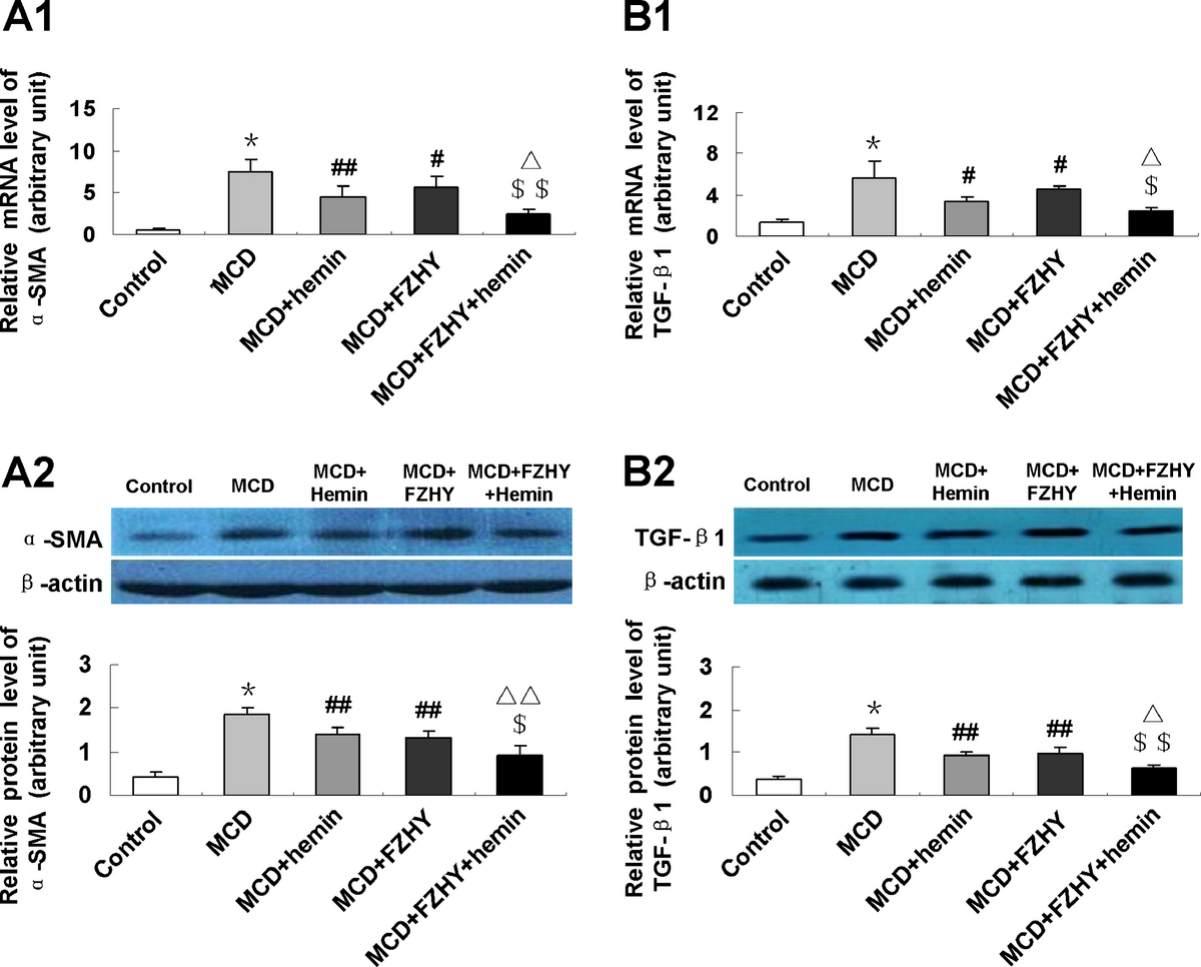
**Effect of FZHY and hemin on hepatic α-SMA and TGF-β1 expression in MCD diet-induced fibrosing steatohepatitis**. mRNA expression of α-SMA (A1) and TGF-β1 (B1) were determined by quantitative real-time PCR; protein levels of α-SMA (A2) and TGF-β1 (B2) were detected by Western blot. Data are expressed as the mean ± SD (n = 6 per group). *P < 0.001, compared with control group; ^#^*P *< 0.05, ^##^*P *< 0.01, compared with MCD group; ^$^*P *< 0.05, ^$$^*P *< 0.01, compared with MCD + FZHY group; ^Δ^*P *< 0.05, ^ΔΔ^*P *< 0.01, compared with MCD + Hemin group.

**Figure 6:**
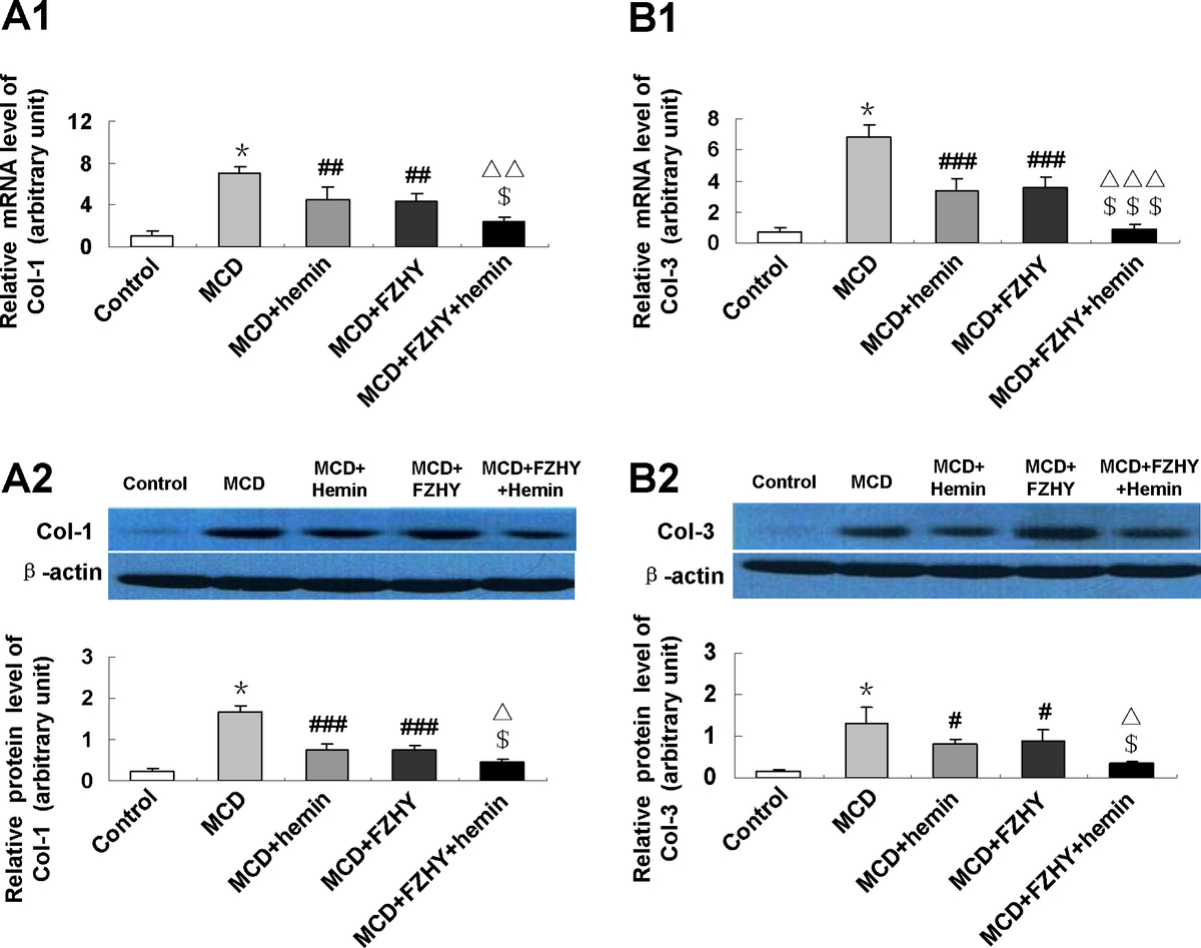
**Effect of FZHY and hemin on hepatic Col-1 and Col-3 expression in MCD diet-induced fibrosing steatohepatitis**. mRNA expression of Col-1 (A1) and Col-3 (B1) were determined by quantitative real-time PCR; protein levels of Col-1 (A2) and Col-3 (B2) were detected by Western blot. Data are expressed as the mean ± SD (n = 6 per group). *P < 0.001, compared with control group; ^#^*P *< 0.05, ^##^*P *< 0.01, ^###^*P *< 0.001, compared with MCD group; ^$^*P *< 0.05, ^$$$^*P *< 0.001, compared with MCD + FZHY group; ^Δ^*P *< 0.05, ^ΔΔ^*P *< 0.01, ^ΔΔΔ^*P *< 0.001, compared with MCD + Hemin group.

To evaluate the mechanism of the effect of FZHY on fibrosing steatohepatitis, we assessed the hepatic expression levels of fibrosis related genes. In MCD feeding mice, mRNA and protein expression of alpha-smooth muscle actin (α-SMA) (Figure 5A), transforming growth factor-β1 (TGF-β1) (Figure 5B), Col-1 (Figure 6A) and Col-3 (Figure 6B) had a marked elevation. The expression of α-SMA (Figure 5A), TGF-β1 (Figure 5B), Col-1 (Figure 6A) and Col-3 (Figure 6B) was significantly down-regulated in the livers of mice treated with FZHY or FZHY plus hemin compared with that administrated with MCD diet alone.

## Discussion

A suitable animal model that can reflect the characteristic metabolic changes and the typical histological lesions of progressive fibrosing steatohepatitis is critical for the evaluation of the therapeutic property of a compound [10]. We used a MCD dietary mouse model for this study, which has been demonstrated typical nutritional fibrotic changes resemble the human beings [10]. Following MCD diet for 8 weeks, mice rapidly and consistently developed a severe pattern of steatohepatitis with typical histopathology, hepatocyte steatosis and necrosis, inflammatory cell infiltration, fibrosis in the pericellular, perisinusoidal and portal area. In accordance with these findings, a significant elevation of serum AST and ALT was presented in MCD diet feeding mice than that in the control. Liver injury could be attenuated by FZHY administration with or without hemin, as evidenced by diminished histological steatosis, inflammation and fibrosis, as well as significantly lowered serum ALT and AST levels. The combination of FZHY and hemin showed a synergetic effect. These results clearly indicated that FZHY modulated hepatic lipid homeostasis and alleviated liver inflammation and fibrosis. In accordance with our finding, Liu et al. showed that FZHY inhibited inflammatory activity, improved the contents of serum albumin and gamma-glutamyltransferase activities [4].

According to the "two hit" hypothesis proposed by Day and James [11], appearance of oxidative stress, overexpression of pro-inflammatory cytokines and mitochondrial dysfunction are the key pathogenic factors involved in development of necroinflammation and ultimately fibrosis and cirrhosis, which are potentially major therapeutic targets in NASH. Oxidative stress, triggered by the accumulation of fatty acids and excessive lipid peroxidation products, leads to mitochondrial dysfunction, hepatic cytochrome CYP2E1 expression, hepatocellular apoptosis and inflammatory cells recruitment, then contributes to stellate cells activation, collagen synthesis and fibrogenesis [12,13]. During the oxidant and anti-oxidant process, HO-1 plays a crucial role in maintaining cellular homeostasis [14]. Under physiologic conditions HO-1 typically occurs at low to undetectable levels in liver but undergoes a rapid transcriptional activation and expresses in both Kupffer cells and hepatocytes as response to noxious stimuli [15]. HO-1 induction is considered to be an adaptive cellular response to survive exposure to environmental stresses. Our previous studies demonstrated that up-regulation HO-1 expression played a vital role in suppressing of oxidative stress, inflammation and fibrosis in various pathological conditions [16-19]. In the present study, we found enhanced oxidative stress in the MCD diet fed mice as demonstrated by significantly increased MDA content, the mRNA and protein expression of CYP2E1. Administration of FZHY significantly reduced hepatic MDA content; suppressed expression of CYP2E1 and induced expression of HO-1 in both mRNA and protein levels, which were concomitant with improved liver histology. These results indicated that FZHY had a protective effect on liver injury through inhibiting oxidative stress by mediating key oxidative stress related factors CYP2E1 and HO-1. In line with our results, decreasing serum ALT, AST and MDA content, improving SOD activity by FZHY had also been observed by others in a chemical induced liver damage animal model [20] and the herbs in FZHY showed a coordinated effect [21].

During the inflammatory process, fibrogensis is part of the wound-healing reaction. The activated Kupffer cells secret pro-inflammatory cytokines such as TNF-α and IL-6, triggers the production of other pro-inflammatory cytokines and fibrogenic factors (e.g. TGF-β1), which futher amplify the profibrogenic actions of HSCs [22,23]. TNF-α and IL-6 are important genes in modulating chemokine and cell adhesion molecule expression, promoting the accumulation of mononuclear leukocytes, stimulating stellate cells activation and collagen synthesis [24,25]. A positive correlation has been found between hepatic TNF-α, IL-6 expression and stage of fibrosis in patients with NASH [26,27]. In this study, we also found the significantly higher levels of hepatic TNF-α and IL-6 in mice fed with MCD diet. However, administration of FZHY significantly reduced the hepatic expression of TNF-α and IL-6 compared with mice fed with MCD diet alone. Moreover, the combination of FZHY and hemin further decreased these cytokines expression. These results suggest that FZHY possesses anti-inflammatory activity by inhibiting the gene expression of pro-inflammatory and pro-fibrotic mediators TNF-α and IL-6, which is beneficial for the treatment of fibrosing steatohepatitis.

In response to oxidative stress, inflammatory cytokines and endothelial matrix alternation caused by chronic liver damage, HSCs undergo a process of transdifferentiation to acquire a myofibroblastic phenotype accompanied by a high expression of α-SMA [28]. HSCs play an unequivocal role in excessive production and accumulation of extracellular matrix in liver fibrosis [29]. It was known that physiologic ECM consists mainly of non-fibrillar collagen, proteoglycans and glycoproteins. Under pathologic conditions the composition of ECM changes into a more fibrillar character with elevated proportion of type I and III collagen, laminin, and fibronectin [30]. We found that the mRNA and protein expression of α-SMA, Col-1 and Col-3 clearly increased in the livers of MCD diet mice, indicating an increase of stellate cell activation and excessive ECM deposition. Administration of FZHY with or without hemin could reduce the expression of α-SMA, Col-1 and Col-3, suggesting that the HSCs activation and collagen synthesis were inhibited by FZHY. Consistent with our findings, previous studies demonstrated that FZHY could inhibit HSC activation [31,32], decrease α-SMA protein expression and Col-1 secretion in liver fibrosis induced by chemical toxin in rats [2,33].

Among the wide variety of cytokines and growth factors secreted by HSC, TGF-β1 plays a predominant role in impacting on collagen metabolism and proliferation. TGF-β1 knockout mice have shown reduced collagen accumulation in response to liver injury compared to that of normal mice [34]. Animal experiments have demonstrated notable anti-fibrotic effect for liver fibrosis using different strategies to block TGF-β1 [35-38]. Moreover, TGF-β1 gene silencing could significantly decreased concentration of pro-inflammatory cytokine TNF-α, suggesting TGF-β1 gene silencing will decrease liver inflammation [39]. In our study, the mRNA and protein expression of TGF-β1 is higher in the livers of mice feeding MCD diet compared with that in the control mice. Treatment with FZHY with or without hemin significantly blunted the expression of TGF-β1. Therefore, anti-fibrotic effect of FZHY was mediated by inhibiting the expression of TGF-β1. This effect was supported by observations from others that FZHY significantly reduced collagen deposition [40], down-regulate the protein expression of α-SMA [2,33,41] and TGF-β1 [31].

In conclusion, the present study provided a novel role of FZHY in protection against nutritional liver fibrosis through suppressing oxidative stress, inflammatory factors and HSCs activation in experimental nutritional steatohepatitis, which were associated with up-regulation of antioxidant gene HO-1, down-regulation of pro-oxidant gene CYP2E1, inflammatory cytokines TNF-α, IL-6, pro-fibrogenic factors α-SMA, TGF-β1, Col-1 and Col-3.

## Abbreviations

FZHY: Fuzheng Huayu recipe; HO-1: Heme oxygenase-1; MCD: Methionine-choline deficient; ALT: Alanine aminotransferase; AST: Aspartate aminotransferase; NAFLD: Nonalcoholic fatty liver disease; NASH: Nonalcoholic steatohepatitis; HSC: Hepatic stellate cell; ROS: Reactive oxygen species; CYP2E1: Cytochrome P4502E1; TNF-α: Tumor necrosis factor-alpha; IL-6: Interleukin-6; IL-10: Interleukin-10; α-SMA: α-smooth muscle actin; TGF-β1: Transforming growth factor beta 1; Col-1: Collagen type I; Col-3: Collagen type III; TBARS: Thiobarbituric acid reactive substances

## Competing interests

The authors declare that they have no competing interests.

## Authors' contributions

YMN designed the research; YHJ, RQW, HMM, LBK, WGR, WCL, SXZ, YGZ, WJW performed the experiments; YHJ and YMN analyzed data; YMN, YHJ and JY wrote the pater. All authors read and approved the final manuscript.
